# UV exposure and melanoma prognostic factors : results from Clinical National Melanoma Registry (CNMR)

**DOI:** 10.1186/1479-5876-12-S1-P3

**Published:** 2014-05-06

**Authors:** Maurizio Montella, Sara Gandini, Carlo Riccardo Rossi, Alessandro Testori, Giuseppe Mastrangelo, Diego Serraino, Mario Budroni, Anna Crispo, Antonio Maria Grimaldi, Paolo Antonio Ascierto

**Affiliations:** 1 Istituto Nazionale Tumori, Fondazione “G. Pascale”, Naples, Italy; 2 Istituto Europeo di Oncologia, Milano, Italy; 3 Department of Molecular Medicine, Padua University, Padua, Italy; 4 Istituto di Ricovero e Cura a Carattere Scientifico Centro di Riferimento Oncologico, Aviano, Italy; 5 University of Sassari, Sassari, Italy

## Background

Previous studies have reported an association between sun exposure and improved cutaneous melanoma (CM) survival. We analysed the association of UV exposure with prognostic factors and outcome in the National Clinical Melanoma Registry.

## Methods

Clinical and socio-demographic features were collected melanoma patients at diagnosis, together with information on sunbed exposure and sunny holidays. Analyses were carried out to investigate the associations between UV exposure and melanoma prognostic factors.

## Results

From December 2010, we retrieved information from 3299 melanoma patients from 39 IMI (Intergruppo Melanoma Italiano) sites geographically representative. 40% of the patients are over 60 years old, 52% are men, 49% live in the north of Italy and 56% are at high educational level (at least high school). 8% of the patients have melanoma familiarity and 55% have fair phenotype (Fitzpatrick skintype I or II). 2% have metastases and 11% lymph-node involvement. 8% have very thick melanoma (Breslow>4 mm) and 21% ulcerated melanoma.

Holidays in the sun five years before CM diagnosis, among non-metastatic patients, were significantly associated with lower Breslow thickness (p=0.003), after multiple adjustment including socio-demographic status, degree of doctor making the diagnosis and residence. Sunbed exposure is not significantly associated with Breslow thickness.

## Conclusions

Holidays in the sun were associated with thinner melanomas. However, these results do not prove a direct causal effect of sun exposure on survival since other confounding factors, such as Vitamin D serum levels, may play a role.
